# Deletion of cardiac fibroblast growth factor-23 beneficially impacts myocardial energy metabolism in left ventricular hypertrophy

**DOI:** 10.1038/s44324-025-00087-w

**Published:** 2025-10-28

**Authors:** Nejla Latic, Arezou Lari, Na Sun, Ana Zupcic, Mhaned Oubounyt, Juliana Falivene, Achim Buck, Martin Hofer, Wenhan Chang, Wolfgang M. Kuebler, Jan Baumbach, Axel K. Walch, Alexander Grabner, Reinhold G. Erben

**Affiliations:** 1 Department of Biological Sciences and Pathobiology, University of Veterinary Medicine, Vienna, Austria; 2 Ludwig Boltzmann Institute of Osteology, Vienna, Austria; 3 Research Unit Analytical Pathology, German Research Center for Health and Environment, Neuherberg, Germany; 4 Institute for Computational Systems Biology, University of Hamburg, Hamburg, Germany; 5 Institute of Physiology, Charité University Medicine, Berlin, Germany; 6 Genomics Core Facility, VetCore, University of Veterinary Medicine, Vienna, Austria; 7 San Francisco VA Medical Center, Department of Medicine, University of California, San Francisco, CA USA; 8 Department of Medicine, Duke University Medical Center, Durham, NC USA

**Keywords:** Metabolism, Metabolomics, Endocrine system and metabolic diseases

## Abstract

Left ventricular hypertrophy (LVH) is associated with increased cardiac expression of fibroblast growth factor-23 (FGF23) in mice and men. To further elucidate the role of cardiac FGF23 in LVH, we specifically ablated *Fgf23* in cardiomyocytes, and employed transverse aortic constriction (TAC) to induce LVH by pressure overload. LVH developed independently of cardiac FGF23, but cardiomyocyte-specific *Fgf23* knock-out (*Fgf23*^CKO^) TAC mice were characterized by ameliorated hypertension and a distinct reduction of cardiac fibrosis, relative to *Fgf23*^fl/fl^ TAC controls. Spatial metabolomics revealed reduced intracellular glucose abundance and lowered cardiac energy charge in *Fgf23*^CKO^ TAC mice, whereas treatment of cultured cardiomyocytes with FGF23 increased intracellular glucose abundance. Spatial transcriptomics showed a downregulation of glucose transporters and glycolytic enzymes, but an upregulation of enzymes involved in fatty acid oxidation in *Fgf23*^CKO^ TAC mice. These findings suggest that reduced cardiac FGF23 signaling promotes cardiac metabolic health by downregulating glucose consumption and favoring fatty acid oxidation.

**Figure:**
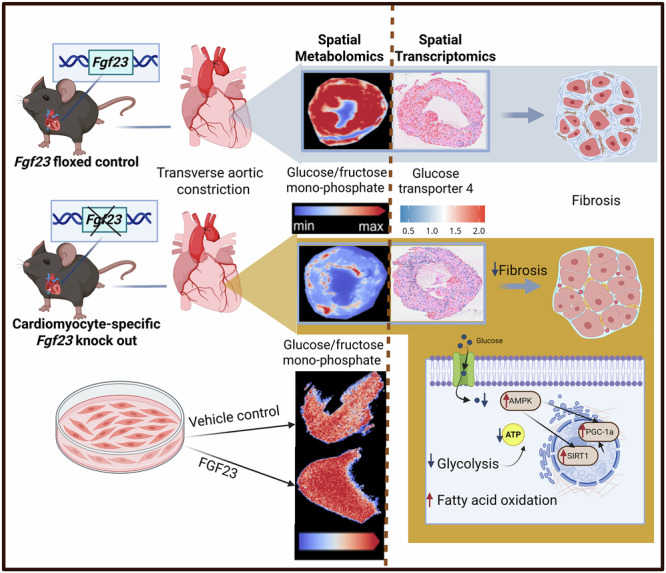
Created in https://BioRender.com

Created in https://BioRender.com

## Introduction

Cardiovascular diseases are the number one cause of mortality worldwide^1^. A plethora of clinical studies have shown that elevated circulating levels of the phosphaturic fibroblast growth factor 23 (FGF23) are associated with left ventricular hypertrophy (LVH), atrial fibrillation, systolic and diastolic dysfunction, as well as heart failure^2–5^. However, the mechanisms underlying this association remain unknown.

FGF23 is a bone-secreted hormone that binds to a receptor complex of FGF receptors (FGFR) and the co-receptor αKlotho to inhibit vitamin D hormone synthesis and proximal tubular phosphate reabsorption in the kidney^6^. In addition, FGF23 has been shown to exert Klotho-independent effects in organs not expressing αKlotho such as the heart under pathological conditions^6,7^. In this context, it was reported that FGF23 causes LVH by binding to FGFR4 on cardiomyocytes and the subsequent activation of the PLCγ/calcineurin/NFAT signaling pathway^8,9^. FGF23 is not only expressed in bone, but also in cardiomyocytes, fibroblasts, and endothelial cells in the heart^10^. It is unclear whether the maladaptive cardiac effects of elevated FGF23 signaling in different disease conditions are mediated through changes in circulating FGF23 or by that produced locally in the heart. It was reported that cardiac FGF23 expression is increased in patients suffering from cardiomyopathies and in murine models of myocardial infarction or LVH^11–15^. Therefore, local FGF23 production could be involved in pathological cardiac remodeling.

Indeed, several independent lines of evidence suggest an important role of upregulated cardiac FGF23 in the development of LVH: first, in mouse models of chronically elevated FGF23 in the circulation, such as *Hyp* mice, excessive concentrations of serum intact FGF23 do not cause LVH in the setting of normal kidney function^16–18^. *Hyp* is the murine homologue of X-linked hypophosphatemia (XLH). Whether XLH patients are characterized by LVH is unclear, as some, but not all, studies associate high FGF23 with LVH in XLH patients^19–21^, suggesting additional contributing factors. Second, we have shown earlier that injection of recombinant FGF23 over a period of five days into mice caused increased blood pressure and heart-to-body weight ratio as an indicator of LVH, but that this prohypertrophic effect of FGF23 was abrogated in Klotho-deficient mice or by co-treatment with a blocker of renal sodium transport^22^. The latter finding suggests that in the absence of a pathological process in the heart, very high blood concentrations of FGF23 may cause LVH by contributing to volume overload through a Na^+^-conserving effect in the kidney, but not through a direct effect on the heart. Based on these findings, it is conceivable that upregulated cardiac FGF23 in the strained heart is a key element of the maladaptive actions of FGF23 on the heart. Consequently, in the absence of a pathological process upregulating FGF23 production in the heart, excessive circulating FGF23 may not cause LVH per se. However, whether this hypothetical scenario is true remains unknown.

To further elucidate the pathophysiological role of locally produced FGF23 in LVH, we used mice with a specific deletion of *Fgf23* in cardiomyocytes in combination with a well-established model of pressure overload-induced cardiac hypertrophy and failure, transverse aortic constriction (TAC). In line with our earlier study in global *Fgf23* knockout mice^14^, we found that local production of FGF23 does not modulate cardiac hypertrophy induced by TAC. However, leveraging spatial genomics technologies in heart cryosections we discovered that lack of cardiac FGF23 resulted in a metabolic switch downregulating glucose and favoring fatty acid consumption in hypertrophic cardiomyocytes.

## Results

### Reduced cardiac FGF23 production neither alters circulating FGF23 levels nor prevents LVH in TAC mice

To examine the pathophysiological role of local FGF23 production in LVH, we employed TAC in combination with the specific ablation of *Fgf23* in cardiomyocytes by crossing *Fgf23*^fl/fl^ mice^23^ with Mlc2v-Cre transgenic mice, specifically expressing Cre in cardiomyocytes. The specificity of Cre expression was confirmed by crossing the Mlc2v-Cre mice with ROSA^mT/mG^ reporter mice (Supplementary Fig. S1A). Cardiomyocyte-specific *Fgf23* knock-out (*Fgf23*^CKO^) mice were born with the expected Mendelian frequency, and their gross phenotype was indistinguishable from that of wild-type (WT) mice. *Fgf23* mRNA levels as measured by qRT-PCR were reduced by 86% in whole hearts of *Fgf23*^CKO^ mice, relative to *Fgf23*^fl/fl^ controls, confirming efficient ablation of Fgf23 in the heart (Supplementary Fig. S1B). Deletion of Fgf23 in cardiomyocytes did not alter serum intact FGF23, calcium, phosphorus, or sodium levels, indicating that there was no perturbation of systemic mineral homeostasis in *Fgf23*^CKO^ mice (Supplementary Fig. S1C–F). Heterozygous Mlc2v-Cre mice lack one functioning allele of the *Mlc2v* gene due to the gene knock-in strategy^24^. To rule out effects of Mlc2v haploinsufficiency, we included heterozygous Mlc2v-Cre transgenic mice (wt/Cre^+^ or wt^+^) as an additional control in studying the effects of TAC. In line with earlier reports in Mlc2v-Cre mice^25^, wt/wt, wt/Cre^+^, and *Fgf23*^fl/fl^ mice showed comparable increases in heart weight-to-body weight ratio (HW/BW), as well as systolic (SP) and mean arterial (MAP) pressures after TAC, indicating a minimal effect of Mlc2v haploinsufficiency per se in the cardiac response to TAC-induced pressure overload (Supplementary Fig. S1G–I).

**Fig. 1:**
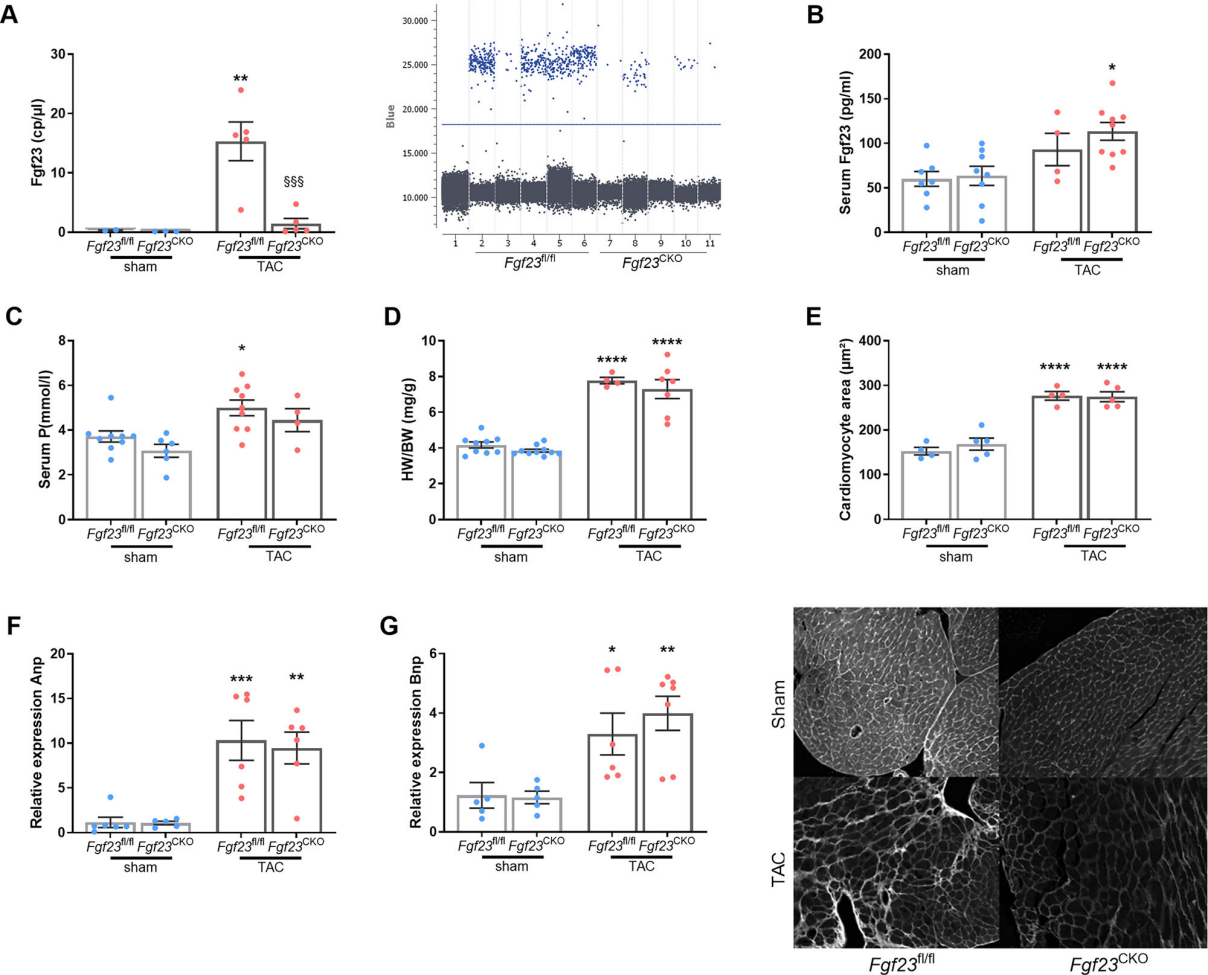
Cardiac production does not contribute to the rise in circulating FGF23, and LV hypertrophy develops independent of cardiac FGF23 after TAC. **A** Left: Cardiac *Fgf23* mRNA expression is increased ~25-fold in *Fgf23*^fl/fl^ mice after TAC as measured by droplet digital PCR (ddPCR) relative to sham controls and *Fgf23*^CKO^ TAC mice (*n* = 3–6). Right: Dot plot of ddPCR of TAC-operated animals. **B** Serum intact FGF23 levels are not different in *Fgf23*^fl/fl^ and *Fgf23*^CKO^ mice, 4 weeks after TAC (*n* = 4–9). **C** Serum phosphorus concentration is higher in *Fgf23*^fl/fl^ mice after TAC, relative to sham controls (*n* = 4–9). **D** Heart/body weight ratio is significantly increased in both genotypes vs. sham controls, 4 weeks after TAC (*n* = 6–9). **E** Upper panel: Quantification of mean cardiomyocyte size after FITC-wheat germ agglutinin (WGA) staining (*n* = 4–5). Lower panel: Representative FITC-labeled WGA -stained sections (scale bar: 100 µm). Relative mRNA expression of markers of hypertrophy, **F** atrial natriuretic peptide (Anp) and **G** brain natriuretic peptide (Bnp) is equally increased in *Fgf23*^CKO^ and *Fgf23*^fl/fl^ TAC mice, relative to sham controls, suggesting that left ventricular hypertrophy (LVH) develops independent of locally produced FGF23 (*n* = 5–7). Bars in (**A**–**G**) represent mean ± SEM for *Fgf23*^fl/fl^ and *Fgf23*^CKO^ mice, 4 weeks after sham or TAC surgery. *p < 0.05, **p < 0.01, ***p < 0.001, ****p < 0.0001 vs. sham control. §§§*p* < 0.001 vs. *Fgf23*^fl/fl^ TAC by one-way ANOVA followed by Student–Newman–Keuls post-hoc test.

In agreement with previous findings reported by us^14^ and others^15^, cardiac *Fgf23* transcript levels were distinctly increased ~25-fold in TAC vs. Sham *Fgf23*^fl/fl^ mice as assessed by droplet digital PCR (ddPCR) (Fig. 1A). In contrast, *Fgf23* transcripts were undetectable in hearts of Sham Fgf23^CKO^ mice and modestly increased by TAC to a level that was less than 10% of the level in *Fgf23*^fl/fl^ control mice subjected to TAC, confirming that cardiomyocytes are the major source of cardiac Fgf23 in response to TAC with only minor contribution from other cell types. To examine Fgf23 expression in fibrotic tissue, we specifically harvested cardiomyocytes and fibrotic areas in situ in cryosections of hearts from TAC *Fgf23*^fl/fl^ mice by laser capture microdissection, and quantified *Fgf23* transcript numbers by ddPCR. We found comparable levels of *Fgf23* transcripts in both cell types (Supplementary Fig. S2), indicating upregulation of Fgf23 transcription in both cardiomyocytes and fibrotic tissue by TAC. The fibrotic response to TAC may contribute to the residual (≈10%) cardiac Fgf23 expression in TAC *Fgf23*^CKO^ mice. However, due to their preponderance in the heart, cardiomyocytes remain the major source of Fgf23 in the heart in response to TAC.

To address the question of whether cardiac FGF23 contributes to the increased circulating FGF23 after TAC, we measured intact FGF23 and C-terminal FGF23 in serum. Interestingly, we found no significant difference in serum intact or C-terminal FGF23 levels between *Fgf23*^fl/fl^ and *Fgf23*^CKO^ mice subjected to TAC, indicating that cardiomyocytes minimally contribute to circulating FGF23 levels (Fig. 1B and Supplementary Fig. S3) in response to TAC. Therefore, the data from the current study support the notion that the increased circulating FGF23 after TAC may be mainly bone derived. It has been reported that blood concentrations of FGF23 increase shortly after cardiogenic shock, making it plausible that the heart directly stimulates skeletal FGF23 production, possibly via proinflammatory cytokines, activation of the renin-angiotensin-aldosterone system (RAAS), or sympathetic activation^26,27^. Despite the increases in circulating FGF23 levels in TAC mice, *Fgf23*^fl/fl^ TAC and *Fgf23*^CKO^ TAC mice were hyperphosphatemic, relative to their Sham controls (Fig. 1C). Therefore, the rise in circulating FGF23 is likely secondary to TAC-induced hyperphosphatemia. The reason for hyperphosphatemia in TAC mice is currently unknown, but may be caused by reduced blood flow to the kidneys. In agreement with the hypothesis that the TAC-induced hyperphosphatemia may be caused by reduced renal blood flow and slightly impaired kidney function, serum creatinine levels tended to be increased both in *Fgf23*^fl/fl^ TAC (13.5 ± 1.0 vs. 11.1 ± 1.2 µmol/l in Sham, *P* = 0.351) and *Fgf23*^CKO^ TAC mice (15.1 ± 1.5 vs. 11.6 ± 0.5 µmol/l in Sham, *P* = 0.101).

Next, we examined the effects of cardiac Fgf23 deletion on the development of TAC-induced LVH. We observed comparable increases in HW/BW ratio between *Fgf23*^fl/fl^ and *Fgf23*^CKO^ TAC mice, 4 weeks post-TAC (Fig. 1D). Similarly, the mean cardiomyocyte area, assessed by histology, and the expression of two markers of hypertrophy, atrial natriuretic peptide (Anp) and brain natriuretic peptide (Bnp), were equally increased in *Fgf23*^CKO^ TAC and *Fgf23*^fl/fl^ TAC mice, compared to the respective Sham controls (Fig. 1E–G). These data indicate that cardiomyocyte-derived FGF23 is not a main inducer of LVH in response to TAC.

### Reduced cardiac Fgf23 expression partially protects against hypertension and fibrosis, but enhances eccentric hypertrophy in TAC mice

**Fig. 2:**
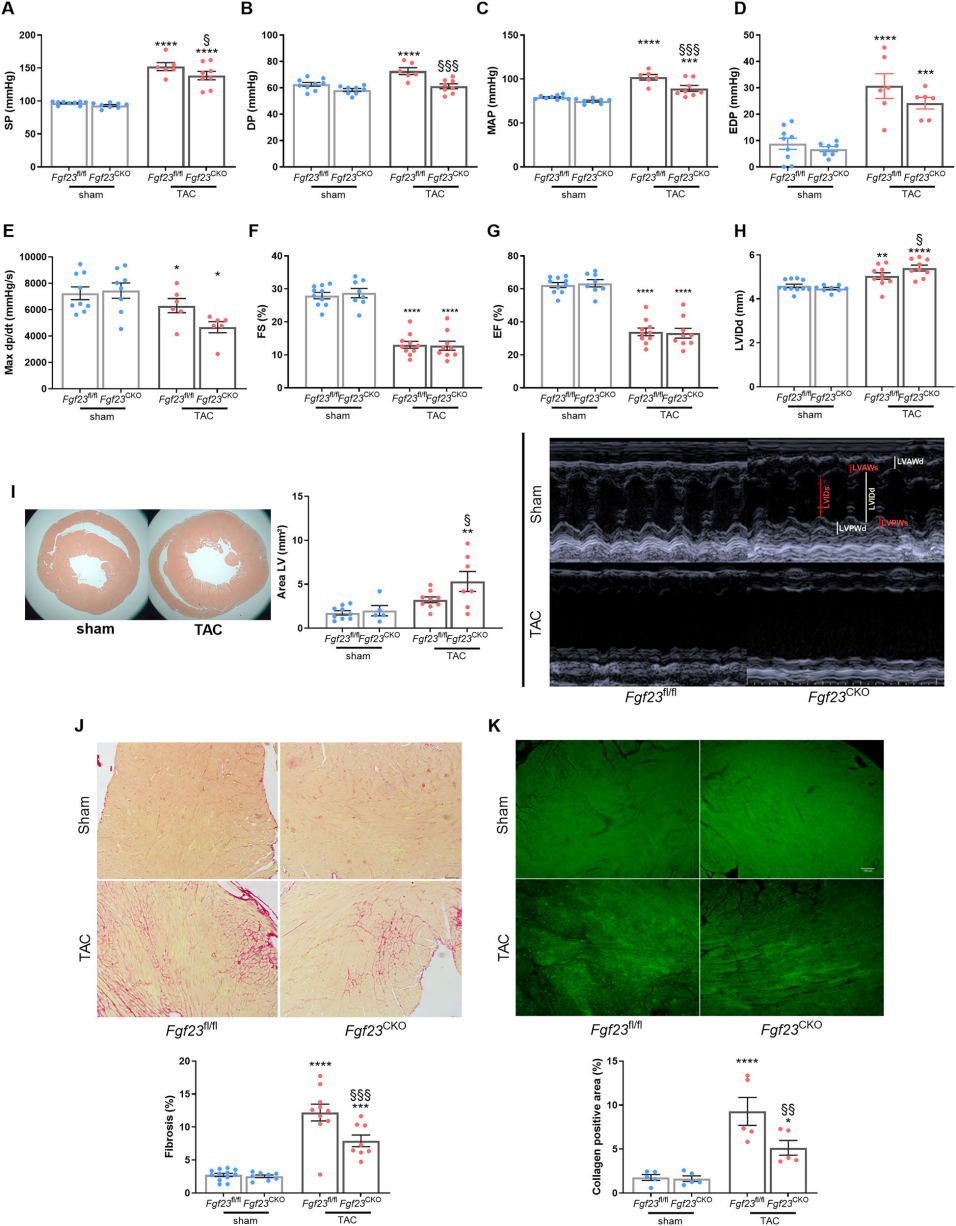
*Fgf23*^CKO^ mice are partially protected against TAC-induced hypertension and fibrosis, but develop a more pronounced eccentric hypertrophy. **A**–**E** Systolic (SP, **A**), diastolic (DP, **B**) and mean arterial blood pressure (MAP, **C**) measured by arterial catheterization are significantly reduced in *Fgf23*^CKO^ mice relative to *Fgf23*^fl/fl^ mice after TAC. Cardiac catheterization revealed a comparable increase in end-diastolic pressure (EDP, **D**) and a decrease in Max dp/dt (**E**) in *Fgf23*^CKO^ mice relative to *Fgf23*^fl/fl^ mice after TAC (*n* = 6–8). **F**–**H** Upper panels: Fractional shortening (FS, **F**), ejection fraction (EF, **G**) and left ventricular internal diameter (LVID, **H**) in diastole measured by echocardiography show unchanged systolic function but an increase in the size of the left ventricle in mice lacking cardiac FGF23, 4 weeks post-TAC (*n* = 9–11). Lower panel: Echocardiography parameters of the left ventricle (short-axis M-mode) (**I**) Left: Representative images of the heart after H&E staining. Right: quantification of the left ventricular area (*n* = 5–9). Top: Representative picrosirius red (PSR, **J**) and immunohistochemical staining for collagen I (**K**) in cardiac paraffin sections (scale bar: 100 µm). Bottom: Quantification of fibrosis revealed that animals lacking FGF23 in cardiomyocytes have strikingly reduced LV fibrotic areas after TAC when compared to *Fgf23*^fl/fl^ controls (*n* = 5–12). Bars in (**A**–**K**) represent mean ± SEM for *Fgf23*^fl/fl^ and *Fgf23*^CKO^ mice, 4 weeks after sham or TAC surgery. *p < 0.05, **p < 0.01, ***p < 0.001, ****p < 0.0001 vs. sham control. §*p* < 0.05, §§*p* < 0.01, §§§*p* < 0.001 vs. *Fgf23*^fl/fl^ TAC by one-way ANOVA followed by Student–Newman–Keuls post-hoc test.

TAC surgery causes a sudden onset of hypertension in the ascending aorta due to the ligation between the brachiocephalic and left common carotid artery. When we measured blood pressure by catheterization via the right carotid artery, we found significantly lower systolic (SP), diastolic (DP) and mean arterial (MAP) pressures in *Fgf23*^CKO^ TAC mice in comparison to *Fgf23*^fl/fl^ TAC mice, 4 weeks post-surgery (Fig. 2A–C). To examine whether the reduced blood pressures were due to a lower blood volume, we measured LV end-diastolic pressure (EDP), and found it was comparably increased by TAC in the *Fgf23*^CKO^ and *Fgf23*^fl/fl^ mice (Fig. 2D), indicating unchanged blood volume and pre-load. Furthermore, LV catheterization revealed a significant decrease in MaxdP/dt, a marker of LV contractility, in *Fgf23*^CKO^ TAC and *Fgf23*^fl/fl^ TAC mice, relative to Sham controls (Fig. 2E). The reduction in LV contractility tended to be more pronounced in *Fgf23*^CKO^ TAC vs. *Fgf23*^fl/fl^ TAC mice, but this effect did not reach statistical significance (Fig. 2E). To assess diastolic function we measured tau, a parameter used to evaluate left ventricular relaxation. We found an increase in tau in *Fgf23*^CKO^ TAC vs. *Fgf23*^CKO^ Sham mice, suggesting impaired diastolic function in these animals (Supplementary Fig. S4).

We next compared changes in functional and morphological parameters that might have contributed to the lower blood pressure observed in the ascending aorta of *Fgf23*^CKO^ mice. Echocardiography revealed no changes in fractional shortening (FS) and ejection fraction (EF) between *Fgf23*^CKO^ TAC and *Fgf23*^fl/fl^ TAC mice. Both parameters were markedly reduced in TAC mice in comparison to sham-operated animals (Fig. 2F, G). However, we found that the TAC-induced increase in diastolic LV internal diameter (LVIDd) in *Fgf23*^CKO^ TAC was larger than that in *Fgf23*^fl/fl^ TAC mice (Fig. 2H, upper and lower panels), which was further confirmed by histomorphometry (Fig. 2I, left and middle panels). Hence, *Fgf23*^CKO^ mice developed a more pronounced eccentric hypertrophy in comparison to *Fgf23*^fl/fl^ mice after TAC. To compare the extent of TAC-induced cardiac fibrosis between *Fgf23*^CKO^ and *Fgf23*^fl/fl^ mice, we quantified collagen content in heart sections stained with picrosirius red and immunostained for collagen-1. Notably, animals lacking FGF23 in cardiomyocytes had strikingly reduced LV fibrotic and collagen 1-positive areas after TAC when compared to *Fgf23*^fl/fl^ TAC controls (Fig. 2J, K).

### Reduced cardiac FGF23 production impacts myocardial energy metabolism after TAC

**Fig. 3:**
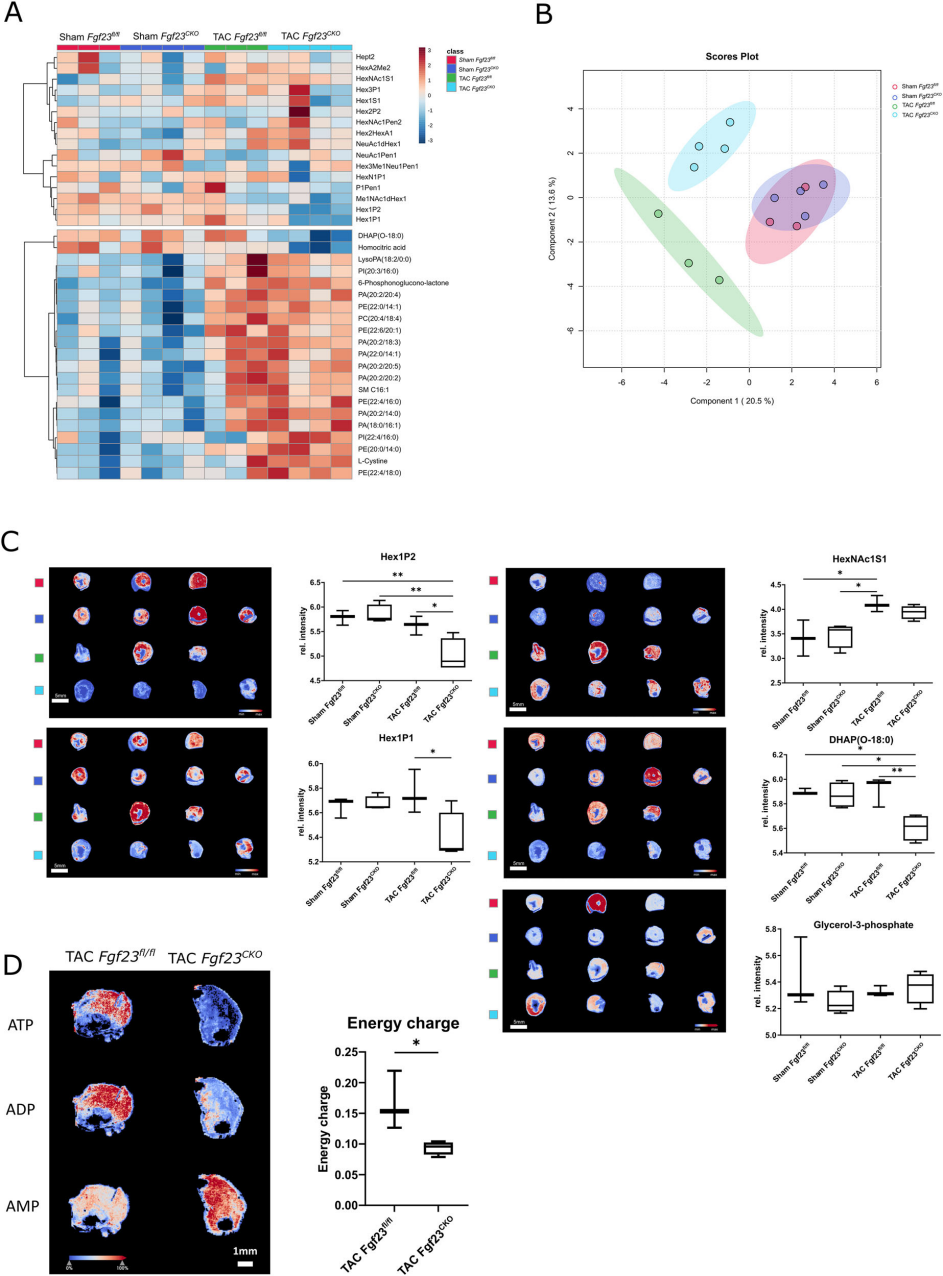
Mass spectrometry imaging reveals impaired cardiac energy metabolism in *Fgf23*^CKO^ mice after TAC. **A** Heatmap of annotated glycan fragments and lipids, and **B** Partial Least Squares Discriminant Analysis (PLS-DA) score plot of the metabolome in heart cryosections reveal clear differences between LVs from *Fgf23*^CKO^ TAC mice and *Fgf23*^fl/fl^ TAC mice. In contrast, LVs from Sham mice of both genotypes show comparable metabolic signatures. **C** Intensity distribution maps and box plots of Hex1P2, HexNac1S1, Hex1P1 and DHAP (O-18:0) show distinct differences between LVs from *Fgf23*^CKO^ and *Fgf23*^fl/fl^ TAC mice, whereas the abundance of glycerol-3-phosphate remains unchanged. *p < 0.05, **p < 0.01, ***p < 0.001 by one-way ANOVA test followed by Tukey’s multiple comparison test. **D** AMP, ADP, and ATP intensity distributions and quantification of energy charge (calculated as the ratio of the concentrations of ATP, ADP, and AMP) demonstrate a pronounced reduction of energy charge in LVs of *Fgf23*^CKO^ vs. *Fgf23*^fl/fl^ TAC mice. *p < 0.05 by two-tailed *t* test. Data in (**A**–**D**) are from 3 to 4 mice per group. Hex hexose, HexNAc N-acetylhexosamine, P phosphate, S sulfate.

To test the hypothesis that the reduction in blood pressure as well as the increased LV chamber size in *Fgf23*^CKO^ TAC mice might be caused by reduced contractile force of the ventricle due to changes in energy metabolism, we employed matrix-assisted laser desorption/ionization (MALDI) imaging mass spectrometry to assess cardiac energy metabolism in situ. This technology enables the simultaneous analysis of hundreds to thousands of analytes in each pixel of a tissue section^28–30^. MALDI imaging in heart cryosections revealed that the metabolic signature of LVs from *Fgf23*^CKO^ TAC mice was clearly different from that of *Fgf23*^fl/fl^ TAC mice,

whereas LVs from Sham mice of both genotypes showed comparable metabolic signatures (Fig. 3A, B). The most striking metabolic differences between LVs from TAC *Fgf23*^CKO^ and *Fgf23*^fl/fl^ mice were the abundance of hexose phosphate metabolites (Hex1P1 and Hex1P2) (Fig. 3A, C). When glucose is taken up by cells, it is immediately phosphorylated by hexokinase. Therefore, the Hex1P1 and Hex1P2 signals reflect intracellular hexose (mainly glucose; MALDI imaging cannot distinguish glucose and fructose due to identical molecular weight) levels. When we calculated cellular energy charge given as (ATP + 0.5 ADP)/(ATP + ADP + AMP) abundance ratio, we found a striking reduction in energy charge in LVs of *Fgf23*^CKO^ TAC vs. *Fgf23*^fl/fl^ TAC mice (Fig. 3D), supporting our hypothesis that lack of cardiac FGF23 production alters energy metabolism in the hypertrophic heart, leading to reduced LV contractile force.

TAC-induced LVH was associated with a profound increase in glycerophospholipid metabolites (C51-PA, C41-PE, C37-PA, etc.), with only a few differences between *Fgf23*^CKO^ and *Fgf23*^fl/fl^ mice (Fig. 3A). The TAC-induced changes in cardiac lipid profiles may help maintain the stability of cell membranes against cardiac stress, and may provide additional sources for energy metabolism in the stressed heart. Interestingly, we observed a reduction in the abundance of dihydroxyacetone phosphate (0–18) (alkyl-DHAP) in TAC mice lacking *Fgf23*, relative to *Fgf23*^fl/fl^ controls (Fig. 3A, C). DHAP is synthesized from glycerol-3-phosphate (G3P). However, cardiac G3P abundance was not different between *Fgf23* ^CKO^ TAC and *Fgf23*^fl/fl^ TAC mice (Fig. 3C).

### FGF23 increases intracellular glucose abundance in cultured rat cardiomyocytes

**Fig. 4:**
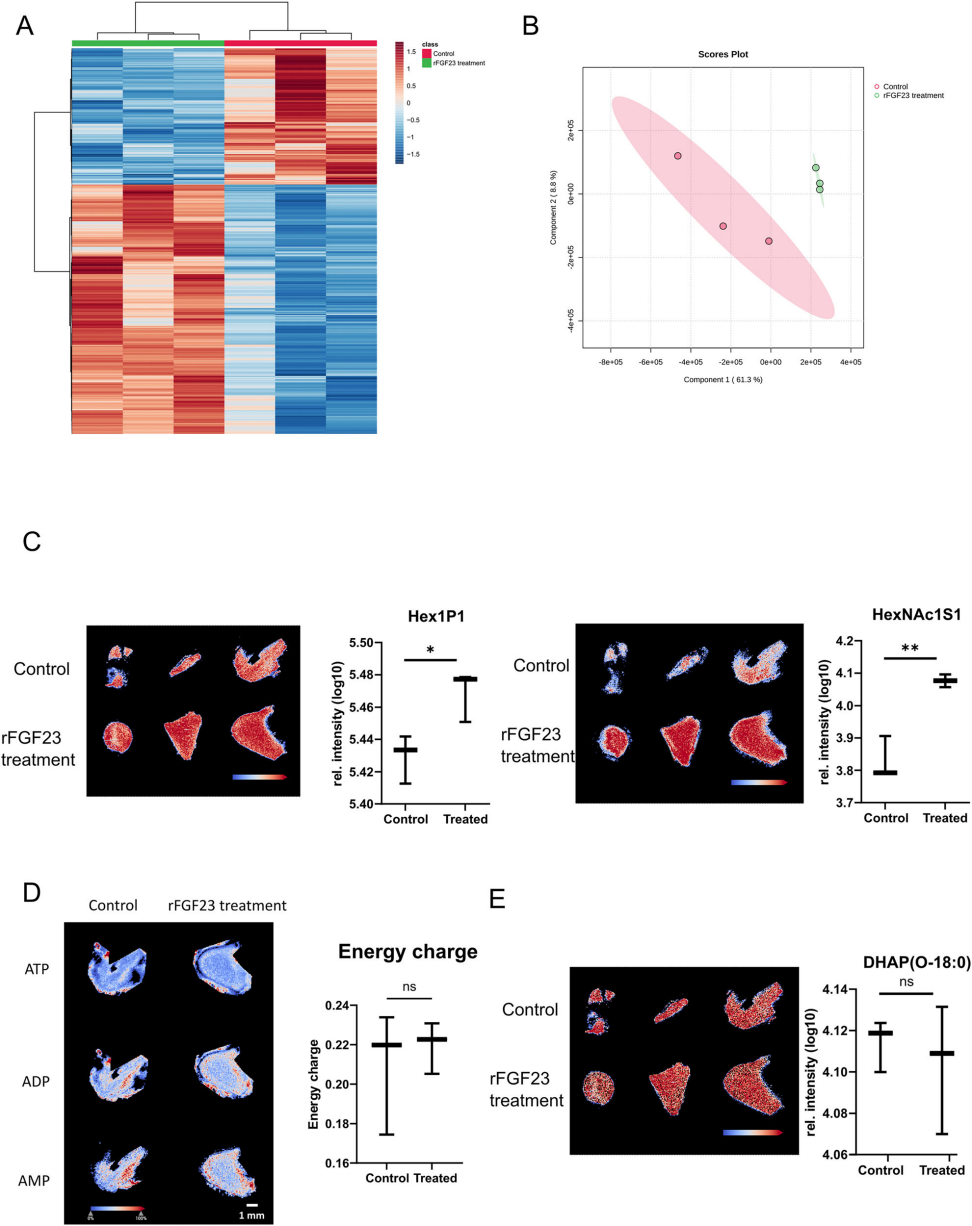
MALDI imaging of cultured cardiomyocytes reveals profound differences in the metabolic signature of FGF23 and vehicle-treated cells. **A** Heatmap of the top 1000 significantly different metabolites and **B** PLS-DA score plot of the metabolome reveals distinct differences in the metabolic signatures of recombinant FGF23 (rFGF23) and vehicle-treated neonatal rat cardiomyocytes. **C** Intensity distribution maps and box plots of Hex1P1 and HexNac1S1 demonstrate an increase in abundance in rFGF23-treated vs. vehicle-treated cardiomyocytes. **D** Energy charge shows no differences between vehicle and rFGF23-treated cells. AMP, ADP, and ATP intensity distributions in vehicle- and rFGF23-treated cells are shown as an example. **E** Abundance of DHAP(O-18:0) remains unchanged under rFGF23 treatment in cultured cardiomyocytes as shown by intensity distribution maps and box plots. Data in (**A**–**D**) are from 3 independent cultures each. *p < 0.05, **p < 0.01 by two-tailed *t*-test. ns not significant, Hex hexose, HexNAc N-acetylhexosamine, P phosphate, S sulfate.

To further test the hypothesis that FGF23 directly modulates energy metabolism in cardiomyocytes, we cultured neonatal rat cardiomyocytes and treated them with recombinant FGF23 (rFGF23) as described previously^9^. Following 48 h of rFGF23 or vehicle treatment, the cultured cells were collected, pelleted, and snap-frozen. MALDI imaging of cryosections of the cell pellets revealed striking differences in the metabolic signatures of rFGF23- and vehicle-treated cells (Fig. 4A, B, Supplementary Fig. S5). As shown in Supplementary Fig. S6, rFGF23 treatment over 48 h induced complex changes in glycan and lipid patterns in cultured cardiomyocytes. Interestingly, FGF23 treatment increased the abundance of intracellular glucose monophosphate (Hex1P1), relative to the vehicle-treated cardiomyocytes (Fig. 4C), confirming our hypothesis that FGF23 increases glucose uptake in hypertrophic cardiomyocytes. However, energy charge was not different between vehicle and FGF23-treated cells (Fig. 4D), a finding that can likely be explained by the lacking energy expenditure in cultered cardiomyocytes, compared to a working heart. In addition, rFGF23 did not alter the abundance of alkyl-DHAP in cultured cardiomyocytes (Fig. 4E).

### Spatial transcriptomics reveal shift in cardiac energy metabolism in response to reduced FGF23 signaling

To gain deeper insight into the metabolic effects of FGF23 signaling in the strained heart i*n vivo*, we performed spatial transcriptomics on cryosections of one heart each from TAC *Fgf23*^CKO^ and *Fgf23*^fl/fl^ mice, using 10x Genomics CytAssist technology. The cross-sections were taken from the middle region of the ventricle, directly adjacent to the region sampled for spatial metabolomics in the same mice. For the transcriptomics analysis, we utilized the Seurat package in R^31^. Differentially expressed genes (DEGs) were identified based on an adjusted p-value of less than 0.05 and a minimum expression proportion (pct) greater than 0.2 in at least one sample.

**Fig. 5:**
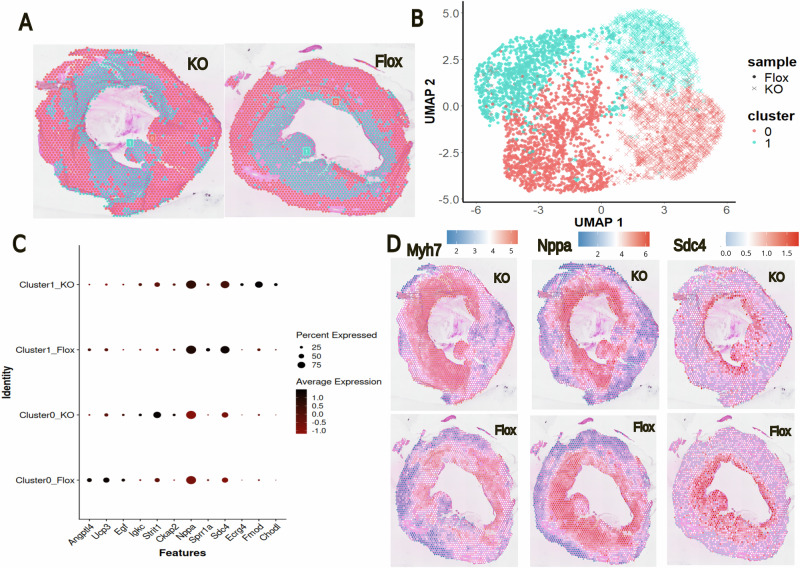
Spatial transcriptomic analysis in the TAC-induced heart failure model in Fgf23CKO and Fgf23fl/fl mice. **A** Spatial cluster mapping in heart cryosections shows two clearly distinct regions: Cluster 0 (red) and Cluster 1 (turquoise). **B** UMAP analysis of gene expression further confirms this separation, with Cluster 1 cells from both genotypes grouping closely in the upper part of the plot, while Cluster 0 cells form a separate group in the lower part. **C** The dot plot highlights the top 12 genes that are most differentially expressed across clusters and genotypes, illustrating key drivers of the differences between them. **D** Spatial expression maps for heavy chain-7 (Myh7), ANP (Nppa), and syndecan-4 (Sdc4) show distinct gene expression patterns across heart sections from *Fgf23*^CKO^ (KO) and *Fgf23*^fl/fl^ (Flox) mice, reflecting both cluster-specific and genotype-specific differences. Color scale indicates log-normalized gene expression per spot (Seurat *LogNormalize*). Blue shows lower expression, white around the average, and red higher expression.

Similar to our earlier report in TAC mice^32^, spatial integration revealed two primary clusters corresponding to inner (cluster 1) and outer (cluster 0) tissue regions in TAC *Fgf23*^CKO^ and *Fgf23*^fl/fl^ mice (Fig. 5A). The outer region includes the outer LV wall and the right ventricle, whereas the inner region includes the LV inner wall and septum (Fig. 5A). UMAP visualization of transcriptomic profiles revealed that spots from the inner and outer layers formed distinct clusters in each genotype (Flox and KO). Interestingly, despite the genotype differences, these clusters were positioned similarly in both groups, indicating that the overall clustering pattern remained consistent (Fig. 5B). This was confirmed by differential gene expression analysis identifying the top three signature genes distinguishing the outer and inner clusters in TAC *Fgf23*^CKO^ and *Fgf23*^fl/fl^ mice (Fig. 5C). In agreement with our earlier work and single cell sequencing studies^32,33^, the inner, more stressed region is characterized by elevated levels of β-myosin heavy chain-7 (Myh7), ANP (Nppa), and syndecan-4 (Sdc4), markers linked to cardiac stress and hypertrophy, relative to the outer, more healthy region (Fig. 5D).

**Fig. 6:**
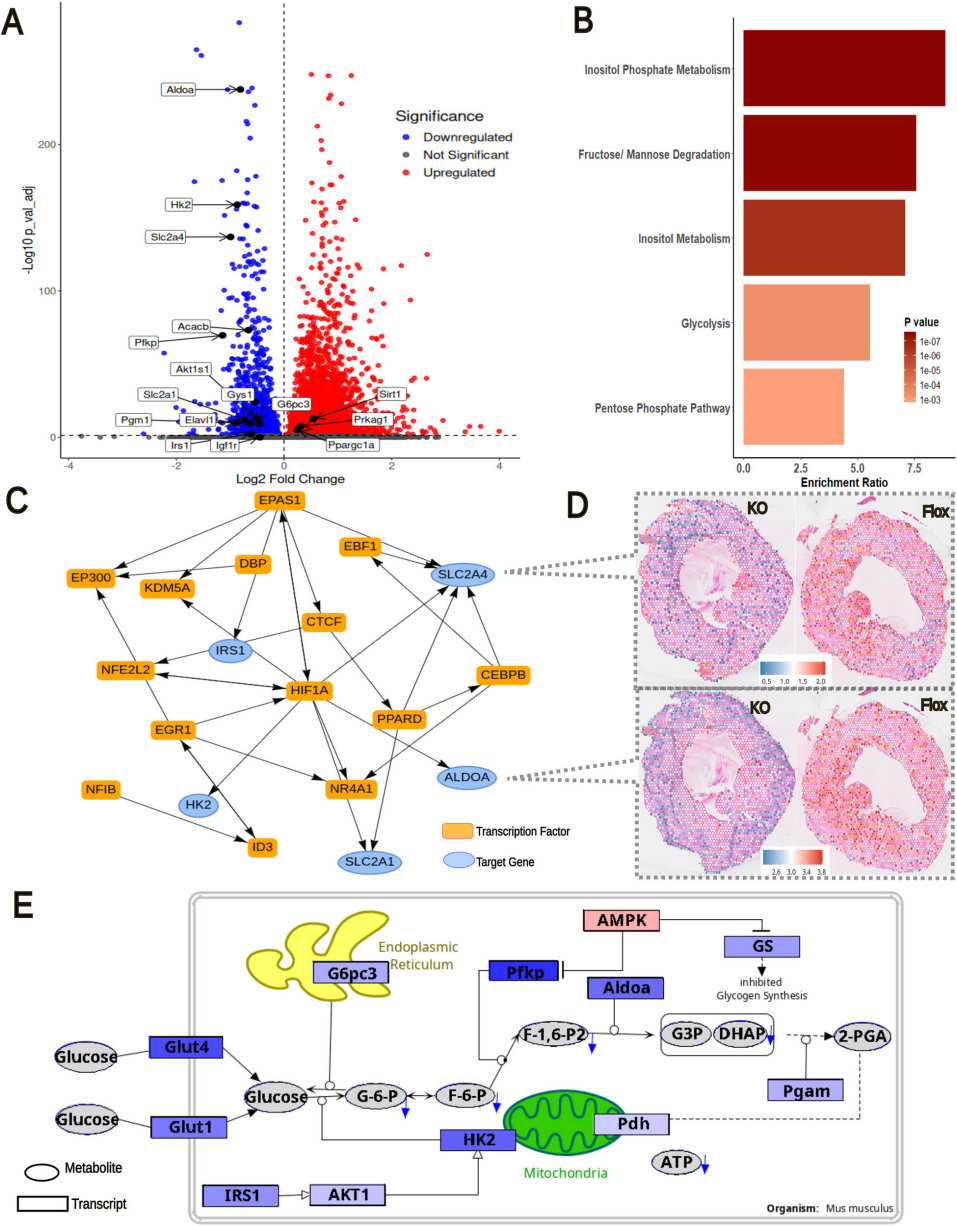
Spatial transcriptomic analysis in hearts of Fgf23^CKO^ and Fgf23^fl/fl^ mice and proposed metabolic mechanisms in the heart of Fgf23^CKO^ mice after TAC, highlighting the alteration in glucose metabolism. **A** Volcano plot of inner-cluster gene expression (KO vs Flox): Many key regulators of glucose metabolism are among the differentially expressed genes, highlighting metabolic alterations in the KO cluster. **B** Top significantly enriched metabolic pathways differentiating KO vs Flox, based on MetaboAnalyst pathway enrichment analysis. **C** Gene regulatory network of key genes involved in glucose metabolism. The network illustrates transcription factors (orange rectangles) and their target genes (blue ellipses) that are significantly dysregulated in the inner layer of heart tissue between Fgf23^CKO^ and Fgf23^fl/fl^ control mice. The arrows indicate the regulation direction: single arrows indicate one-way regulatory effects; double edges indicate mutual effects. **D** Spatial distribution of Slc2a4 (GLUT4) and Aldoa (Aldolase A) expression, key glucose metabolism–related DEGs. Color scale indicates log-normalized gene expression per spot (Seurat LogNormalize). Blue shows lower expression, white around the average, and red higher expression. **E** Proposed metabolic mechanisms in cardiomyocytes of Fgf23^CKO^ mice, highlighting the alterations in glucose metabolism. Pathway visualization was created with PathVisio^61^. Red indicates upregulated transcripts and blue indicates downregulated transcripts. Color intensity corresponds to the magnitude of log-fold change (darker = larger change).

For subsequent analysis, we focused on the inner, more stressed region. We observed major transcriptional regulation as a consequence of reduced FGF23 signaling in the inner heart region of TAC *Fgf23*^CKO^ vs. *Fgf23*^fl/fl^ mice (Fig. 6A). Among the DEGs in the TAC *Fgf23*^CKO^ heart, several key downregulated genes involved in energy metabolism were identified, including Slc2a1 (GLUT1) and Slc2a4 (GLUT4), which are essential for glucose uptake, hexokinase (Hk2), the enzyme essential for phosphorylation of intracellular glucose, key enzymes in glycolysis such as Pfkp (phosphofructokinase) and Aldoa (aldolase A), insulin signaling components such as IRS1 and AKT1, as well as Acacb (Acetyl-CoA Carboxylase Beta), a crucial regulator of lipid metabolism (Fig. 6A). Conversely, genes promoting fatty acid oxidation were upregulated. The key cellular energy sensor AMP kinase (AMPK, Prkag1) showed increased expression in the TAC *Fgf23*^CKO^ heart (Fig. 6A). After activation, AMPK phosphorylates and inhibits Acacb, allowing for increased transport of long-chain fatty acids into mitochondria for β-oxidation via carnitine palmitoyltransferase 1b. In addition, Sirt1 (Sirtuin-1) and PGC-1α (peroxisome proliferator-activated receptor gamma coactivator 1α, Ppargc1a) were upregulated in the TAC *Fgf23*^CKO^ heart (Fig. 6A). AMPK and Sirt1 activate PGC-1α, promoting mitochondrial biogenesis, enhancing lipid oxidation, and improving mitochondrial efficiency^34^.

Metabolic pathway enrichment analysis of the spatial metabolomics data, performed using MetaboAnalyst 6.0^35^, revealed the regulation of key pathways related to metabolic processes in the TAC *Fgf23*^CKO^ heart, including inositol phosphate metabolism, fructose-mannose degradation, inositol metabolism, glycolysis, and pentose phosphate pathway (Fig. 6B). We used DiNiro^36^ to analyze gene regulatory networks (GRNs) potentially involved in the downregulation of Slc2a1, Slc2a4, Hk2, Aldoa, and IRS1 (Fig. 6C). Figure 6C includes only transcription factors (rectangles) dysregulated at the transcriptional level in the inner region of the TAC *Fgf23*^CKO^ heart. It is evident that the transcriptional regulation of Slc2a1, Slc2a4, Hk2, Aldoa, and IRS1 is complex, and is likely not driven by a single transcription factor. The spatial distribution of Slc2a4 and Aldoa in *Fgf23*^CKO^ vs. *Fgf23*^fl/fl^ TAC hearts is depicted in Fig. 6D.

**Fig. 7:**
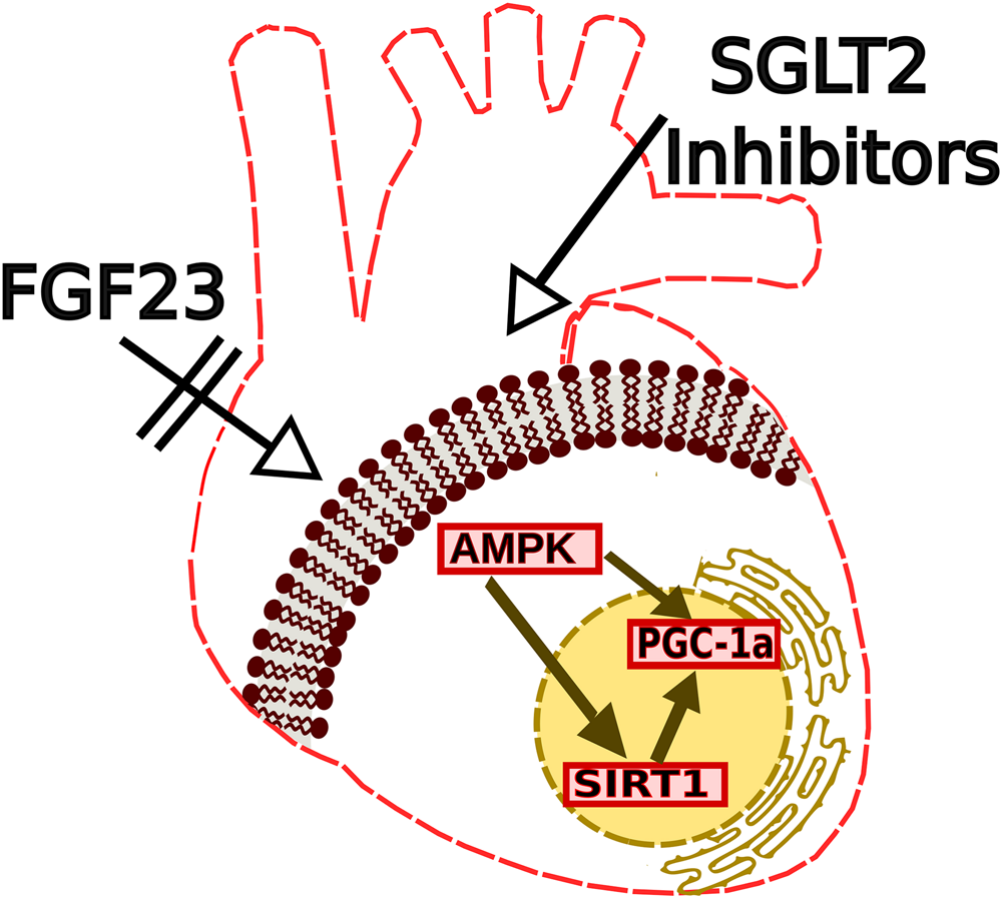
Proposed common cardioprotective pathway of SGLT2 inhibitors and of reduced FGF23 signaling. Sodium glucose transporter-2 (SGLT2) inhibitors have shown protective effects in left ventricular hypertrophy, potentially through activation of the AMPK–SIRT1–PGC-1α (AMP kinase – sirtuin-1 - peroxisome proliferator-activated receptor gamma coactivator 1α) pathway. Interestingly, lack of FGF23 signaling in cardiomyocyte-specific *Fgf23* knockout mice led to an upregulation of the same pathway in TAC-induced cardiac hypertrophy. This overlap suggests a shared mechanism that may underly the cardioprotective effects observed in both settings.

The scheme shown in Fig. 6E summarizes the metabolic changes found by spatial transcriptomics and metabolomics in the TAC *Fgf23*^CKO^ heart, focusing on glucose metabolism. The scheme is based on the integration of the spatial transcriptomics and metabolomics data from the same two mice with the help of the MALDIquant package in R^37^. Transcriptomic changes are shown by rectangles, metabolic changes by ellipses. The TAC *Fgf23*^CKO^ heart was characterized by a downregulation of genes related to glucose uptake and utilization. Both GLUT1 and GLUT4, critical glucose transporters, were downregulated in the TAC *Fgf23*^CKO^ heart, reducing glucose entry into cardiomyocytes and limiting ATP production. In addition, transcript levels of key glycolytic enzymes such as Hk2, Pfkp, and Aldoa, were suppressed, similar to genes involved in glycogen synthesis (glycogen synthase, GS) and insulin signaling (IRS1 and AKT1). In line with the transcriptomic changes in the TAC *Fgf23*^CKO^ heart, metabolites associated with glycolysis and energy production were downrgulated, such as glucose-6-phosphate, fructose-1,6-bisphosphate, DHAP, and ATP (Fig. 6E). Conversely, genes involved in lipid utilization such as AMPK, Sirt1, and PGC-1α were increased in the TAC *Fgf23*^CKO^ vs. *Fgf23*^fl/fl^ heart (Fig. 6A). The upregulation of AMPK, Sirt1, and PGC-1α expression found in the TAC *Fgf23*^CKO^ heart may occur in response to energy stress, providing a compensatory mechanism for the reduction in glucose utilization in the inner heart region of *Fgf23*^CKO^ mice. The upregulation of AMPK, Sirt1, and PGC-1α expression may enhance lipid oxidation and mitochondrial efficiency, thereby helping to maintain ATP production, to reduce oxidative stress, and to support cardiomyocyte survival. Collectively, the changes observed in the heart of the TAC *Fgf23*^CKO^ mouse reflect a metabolic switch, downregulating glucose consumption while at the same time favoring fatty acid oxidation. It is interesting to note in this context that this mechanism aligns with the cardioprotective effects observed with sodium glucose transporter-2 (SGLT2) inhibitors^38^. Hence, the lack of FGF23 signaling in the stressed heart mimics the protective cardiometabolic effects of SGLT2 inhibitors (Fig. 7).

## Discussion

It is known in mice and men that LVH is associated with an upregulation of FGF23 transcription in the heart^15,39^. However, the potential pathophysiological role of paracrine FGF23 signaling in LVH is unclear. Our study in cardiomyocyte-specific *Fgf23* conditional knockout mice subjected to TAC as a pressure overload-induced LVH model has shown that the development of LVH occurs independent of cardiac FGF23. This finding is in line with a recent report in a similar animal model^15^ and our earlier findings in global *Fgf23* knockout mice^14^. However, using spatial metabolomics in heart cryosections, we made the discovery that the lack of local FGF23 secretion profoundly reduced intracellular glucose abundance and energy charge in hypertrophic cardiomyocytes. Conversely, treatment of cultured cardiomyocytes with rFGF23 stimulated the abundance of intracellular glucose, with the caveat that it is not possible to distinguish endocrine from paracrine effects in these experiments. Spatial transcriptomics revealed that lack of cardiac FGF23 resulted in a metabolic switch, downregulating glucose and favoring fatty acid consumption. Hence, in line with a previous report in a murine CKD model^40^, our study has linked paracrine FGF23 secretion in the heart to glucose and energy metabolism in hypertrophic cardiomyocytes, uncovering a new biological role of FGF23 in the heart.

It is known that under pathological conditions such as pressure or volume overload the heart shifts from fatty acid to glucose metabolism for more efficient ATP production per O_2_ consumed^41^. This metabolic switch is associated with an upregulation of the GLUT1 abundance in the plasma membrane^42^. Based on our finding that intracellular phospho-glucose abundance and energy charge were reduced in the heart of TAC *Fgf23*^CKO^ mice, we hypothesize that the profound upregulation in cardiac FGF23 production after TAC is part of a hypertrophy program in cardiomyocytes in which FGF23 serves as a paracrine mediator to increase glucose uptake. Hence, based on our data, the main biological function of upregulated paracrine FGF23 secretion as part of the hypertrophy program may be to aid in the metabolic switch from fatty acid to glucose metabolism. It is well established that FGF23 acts on cardiomyocytes via Klotho-independent FGFR4 signaling^9,40^. Therefore, we hypothesize that the FGF23-induced upregulation of glucose uptake in hypertrophic cardiomyocytes may be FGFR4-mediated. It is known that GLUT1 expression is regulated by activation of the RAS-MAPK pathway^43^, making it conceivable that GLUT1 may be a downstream target of FGFR4 signaling.

It was serendipitously found that SGLT2 inhibitors have a protective role in patients with LVH^44^. The heart does not express SGLT2. Hence, the mechanism of action of SGLT2 inhibitors in LVH is still not entirely clear. However, it has been shown recently in heart failure patients that SGLT2 inhibitors increase ketone body-related and short-/medium-chain acylcarnitine metabolites in the blood^45^. In addition, experiments in porcine heart failure models have shown that SGLT2 inhibitors alter cardiac metabolism by increasing fatty acid and ketone body oxidation, and decreasing glucose consumption^46^. Therefore, the beneficial effect of SGLT2 inhibitors in LVH may be based on a metabolic switch in the hypertrophic myocardium, decreasing glucose consumption and increasing utilization of ketone bodies and fatty acids^38,47^. Similarly, our study has shown that lack of cardiac FGF23 signaling mimics the cardioprotective effects of SGLT2 inhibitors by downregulating glucose and upregulating lipid utilization in hypertrophic cardiomyocytes. Considering these findings, it is tempting to speculate that our data may provide an explanation for why excessive FGF23 is associated with untoward outcomes in patients with LVH: the FGF23-mediated increase in cardiac glucose consumption may be detrimental for the failing heart.

An interesting observation in our study was that TAC-induced fibrosis was distinctly ameliorated in *Fgf23*^CKO^ compared with *Fgf23*^fl/fl^ mice. In agreement with our data, studies in heart failure patients found a correlation between increased levels of circulating FGF23 and LV fibrosis^48^. It is conceivable that cardiomyocyte-derived FGF23 may promote the local activation of fibroblasts under pathological conditions. In this context, it has been reported that FGF23 augmented pro-fibrotic signaling in injury-primed fibroblasts via activation of FGFR4 in the kidney^49^. A similar mechanism may occur in the heart. Moreover, it is known that angiotensin II and aldosterone directly stimulate collagen synthesis. Recently, in vitro studies in neonatal rat cardiomyocytes, fibroblasts and adult mouse cardiac fibroblasts found that local activation of RAAS in the heart can stimulate pro-fibrotic crosstalk between cardiomyocytes and fibroblasts, and that FGF23 might promote fibrosis via activation of β-catenin^50,51^.

In agreement with a pro-fibrotic action of paracrine FGF23 in the heart, MALDI imaging revealed a decrease in alkyl-DHAP in *Fgf23*^CKO^ mice when compared to *Fgf23*^fl/fl^ controls after TAC. Alkyl-DHAP is an octadecanoyl derivative of DHAP and is strongly reduced in fibroblasts derived from patients suffering from Zellweger syndrome^52^. Furthermore, alkyl-DHAP is essential for generation of plasmalogens^53^. A recent study in patients suffering from systemic sclerosis (SSc), an autoimmune disease with fibrosis of the skin and/or internal organs has shown a significant increase in plasmalogen production in these subjects when compared to controls, suggesting that plasmalogens may also play a crucial role in fibrotic changes after TAC^54^.

An alternative explanation for the reduced cardiac fibrosis in *Fgf23*^CKO^ mice in our study may be an indirect effect mediated through energy metabolism. In this explanatory scenario, lower energy charge in hypertrophic cardiomyocytes would lead to reduced contractile forces, lower systolic blood pressure, which in turn may lead to lower LV wall tension and to reduced fibrosis. This scenario would explain all aspects of the phenotype of *Fgf23*^CKO^ TAC mice observed in our study, namely reduced blood pressure, augmented LV dilatation, and ameliorated fibrosis. In addition, this model is supported by the fact that a significant reduction in cardiac fibrosis after TAC was not seen in an independently established murine model of cardiac *Fgf23* deletion with a slightly different experimental setting^15^, and that rFGF23 treatment did not regulate alkyl-DHAP in cultured rat neonatal cardiomyocytes in our study. Hence, we believe that the protection against cardiac fibrosis in *Fgf23*^CKO^ TAC mice observed in our study is mediated mainly indirectly through reduced glucose and ATP concentrations in hypertrophic cardiomyocytes of mice lacking cardiac FGF23.

Taken together, our study has uncovered that local production of FGF23 in the heart regulates energy metabolism in hypertrophic cardiomyocytes. Based on the therapeutic efficacy of SGLT2 inhibitors in LVH, our findings may have major translational implications: FGF23 signaling in the heart may be a drug target for the regulation of energy metabolite flux in stressed cardiomyocytes.

## Methods

### Animals

All animal procedures were approved by the Animal Welfare Committee of the Austrian Federal Ministry of Education, Science and Research and were undertaken in accordance with prevailing guidelines for animal care and welfare (permit number BMWF-68.205/0153-WF/V/3b/2014). *Fgf23* floxed mice were generated as described previously^23^. To inactivate the *Fgf23* gene specifically in cardiomyocytes, we bred *Fgf23*^fl/fl^ mice carrying loxP sequences surrounding exon 1 to mice expressing Cre recombinase under the control of the ventricle-specific *Mlc2v* promoter, to obtain *Fgf23*^fl/fl^/*Mlc2v*^Cre/wt^ mice, hereafter referred to as cardiomyocyte-specific *Fgf23* knockout mice (*Fgf23*^CKO^). The mice were genotyped by PCR on genomic DNA isolated from ear clips. Animals were kept on 24 °C with a 12/12-h light/dark cycle. They were fed a normal mouse diet (Sniff, Soest, Germany), and had access to food and tap water ad libitum. At necropsy, the mice were exsanguinated from the abdominal vena cava under general anesthesia with ketamine/medetomidine (100/0.25 mg/kg i.p.) for serum collection.

### Transverse aortic constriction

Animals were subjected to either transverse aortic constriction (TAC) or sham surgery. For the procedure, mice were under general anesthesia induced by intraperitoneal injection of ketamine/medetomidine, followed by endotracheal intubation. For this purpose, a small animal ventilator (Model 849, Harvard Apparatus) and ventilation with a tidal volume of 200 µl and frequency of 200 breathing cycles per min was used. Subsequently, a sternotomy was performed, and the transverse aorta was visualized. The TAC surgery involved placing a 6-0 silk suture ligature over a 27 G needle between the brachiocephalic and left common carotid artery, followed by prompt removal of the needle. Sham animals underwent the same procedure without the aortic ligation. In order to prevent pain and infection post-surgery, animals were injected with a combination of analgesics (buprenorphine 0.25 mg/kg s.c.) and antibiotics (enrofloxacin, 10 mg/kg s.c) for four days. After that, body weight of the animals was checked on a weekly basis. If at any time individual animals lost more than 20% of their initial body weight, they were killed prior to the end of the experiment. All other animals were killed by exsanguination from the abdominal vena cava, 4 weeks post-surgery.

### Biochemical analysis

Serum sodium, phosphate, and calcium were measured using a Cobas c111 analyzer (Roche Diagnostics). Serum intact and C-terminal FGF23 were measured using commercially available ELISAs (Kainos and Quidel, respectively) according to the manufacturer’s instructions.

### Echocardiography

Echocardiography was performed four weeks post-surgery using a 14 MHz linear transducer (Siemens Acuson s2000) for evaluation of cardiac function. Mice were under 1% isoflurane anesthesia with a stable body temperature of 37 °C. M-mode in short-axis at the level of the papillary muscles was used to evaluate left ventricular thickness, ejection fraction, fractional shortening and internal diameters in systole and diastole. Pulsed wave Doppler in the apical four chamber view was used to assess diastolic function. At least four cardiac cycles were analyzed for each parameter.

### Central arterial and cardiac pressure measurements

Central arterial pressure was measured by inserting a micro-tip catheter (1.4 Millar instruments) into the ascending aorta via the right carotid artery under 1.5% isoflurane anesthesia. The catheter was then further advanced into the left ventricle to obtain cardiac pressure parameters. Traces were recorded for at least three minutes and analyzed via LabChart 7 software.

### Histological evaluation

Hearts were fixed in 4% paraformaldehyde, paraffin-embedded, and cut into 5 µm sections. Fibrotic tissue was visualized using picrosirius red and immunohistochemical staining for collagen I in cardiac paraffin sections. Immunohistochemistry (IHC) was used to detect Col1 in the myocardium using an anti-mouse rabbit polyclonal antibody (Proteintech, AB2082037). Antigen retrieval was performed by heating the de-paraffinized cardiac sections to 100 °C for 20 min in citrate buffer (pH 6). Sections were then treated with 0.1% Triton X-100 for 20 min at room temperature to permeabilize cell membranes, and for further 60 min with blocking solution containing 10% goat serum to prevent unspecific antibody binding. Primary antibody against Col1a (rabbit polyclonal IgG, 1:400 in blocking solution, Proteintech) was incubated for two hours at room temperature. After washing, secondary anti-rabbit Alexa Fluor 488 (Invitrogen) antibody was incubated for 1 h at room temperature. All sections were imaged on Zeiss Imager Z2 microscope. Fibrosis was quantified using ImageJ software and was expressed as the ratio of PSR-stained area to total muscle area of the left ventricle and septum or as percentage of Col1 positive area. For the analysis of cardiomyocyte size, cardiac sections were stained with FITC-labeled wheat germ agglutinin (Sigma L4895). At least ten random areas of the heart were measured, and only cardiomyocytes with well-defined borders and visible nuclei were used. Images were obtained by the Zeiss LSM 880 Airyscan confocal microscope and analyzed using semi-automated Image J software. All histological images were analyzed by two independent investigators in a blinded manner.

### RNA isolation and quantitative RT-PCR

Total RNA was isolated from snap-frozen tissue after homogenization in a 24 FastPrep machine using TRI Reagent. The nucleic acid concentration and integrity were determined by electrophoresis (Agilent TapeStation). Only samples that had a RIN value above seven were used. Two µg of RNA was transcribed into cDNA using the High Capacity cDNA Reverse Transcription Kit (Applied Biosystems). Quantitative RT-PCR was performed on a qTOWER3/G qPCR device (Analytic Jena). The qPCR performed in a volume of 15 μl was composed of 1 × PCR buffer B2 (Tris-HCl, (NH_4_)_2_SO_4_ and Tween-20; Solis Biodyne, Tartu, Estonia), 3.5 mM MgCl_2_, 200 µM dNTP mix (Solis Biodyne), 250 nM of each primer and either 200 nM hydrolysis probe or 0.4 x EvaGreen (Biotium), 1 U HOT FIREPol® DNA polymerase (Solis Biodyne) and 2 μl template DNA. For mouse primer sequences see Supplementary Table 1. Cycling conditions consisted of an initial 15-min incubation step at 95 °C for polymerase activation and template denaturation, followed by 45 cycles of 95 °C denaturation for 15 s and 60 °C annealing and elongation for 60 s. All samples were measured in triplicates and normalized to two housekeeping genes (*Dpm1* and *Txnl4a*). qPCR results were obtained and primarily evaluated with the software qPCRsoft 4.1 (V4.1.3.0), and then analyzed using the standard delta delta Cq method.

### Laser Capture Microdissection (LCM)

Hearts were snap-frozen in liquid nitrogen with OCT compound (Sakura Finetek) and stored at −80 °C until used. Eight-μm-thick cryosections were cut on a cryotome (Leica CM1950), and placed on membrane slides (Carl Zeiss). Cryosections were quickly stained with HistoStain (Arcturus), and cardiomyocytes and fibroblasts (~10,000 cells per sample each) were harvested using a Leica LMD7 LCM system as described previously^55^.

### RNA Isolation and droplet digital PCR (ddPCR)

RNA from LCM samples was isolated using the RNeasy Micro Kit (Qiagen). The yield and integrity were assessed using micro-capillary electrophoresis (Agilent 2100 Bioanalyzer System), and only samples with RIN values above 6.5 were used. A minimum of 2 ng of RNA was transcribed into cDNA using SuperScript IV VILO Master Mix (ThermoFisher). A primer-probe set was designed for the gene of interest and PCR reactions were carried out using PerfeCTa Multiplex qPCR ToughMix (Quanta Bio). For mouse primer sequences see Supplementary Table 1. For precise and absolute quantification of small amounts of the gene of interest, digital PCR was performed on a Naica Crystal Digital™ PCR System (Stilla Technologies), using Sapphire Chips (Stilla Technologies). The mastermix contained 1× PerfeCTa Multiplex qPCR ToughMix (Quanta Bio) with 100 nM fluorescein, 800 nM of each primer and 250 nM hydrolysis probe and up to 16 µl template cDNA. After the droplet generation, the following run protocol was applied: 1 × 95 °C for 10 min, 45 × 95 °C for 10 sec and 60 °C for 40 sec. The finished chips were read out on the Naica Prism 3 reader and then the so gained data was analyzed in the Crystal Miner software (V2.4.0.3; Stilla Technologies). Data are presented as number of copies of target DNA in the starting sample.

### Culture of neonatal rat cardiomyocytes

Neonatal rat cardiomyocytes were cultured as described^9^. In brief, the pups were euthanized via rapid decapitation and the hearts were quickly excised and placed in ice-cold calcium- and magnesium-free Hank’s Balanced Salt Solution (HBSS). The hearts were then minced and digested with 50 μg/mL trypsin at 4 °C for 18–20 h. Soybean trypsin inhibitor in HBSS was added, and the tissue was further digested with collagenase (in Leibovitz L-15 medium) under slow rotation (15 rpm) at 37 °C for 45 min. Cells were released by triturating the suspension 20 times with a standard 10 ml plastic serological pipette and filtering it twice through a cell strainer (70 μm, BD Falcon). Cells were incubated at room temperature for 20 min and spun at 75 x g for 5 min. The cell pellet was resuspended in plating medium Dulbecco’s Modified Eagle Medium (DMEM) with 17% Media 199 (Invitrogen, MA, USA), 15% fetal bovine serum (FBS; Invitrogen) and 1% Antibiotic-Antimycotic (Cat.no 15240096, Gibco). The cell suspension was then plated onto culture plates and incubated at 37 °C and 5% CO_2_ for at least 2 h. Non-myocytes were then removed by differential plating and myocytes were collected and counted using an automated cell counter (Bio-Rad). Myocytes were left undisturbed in plating medium and then cultured in maintenance medium (DMEM with 20% Media 199, 1% insulin-transferrin-sodium selenite solution [ITS; Sigma-Aldrich] and 1% Anti/Anti). After 4 days, isolated cardiac myocytes were cultured in maintenance medium in the presence of recombinant murine FGF23 (at 100 ng/mL) and Heparin (0.2 USP/mL) for 48 h.

### MALDI mass spectrometry imaging

Cryosections of 8-μm thickness were prepared using a cryotome (Leica CM1950). The tissue sections were thaw-mounted on indium tin oxide-coated conductive glass slides (Bruker Daltonik). The tissue sections were spray-coated with 10 mg/mL of 9-aminoacridine hydrochloride monohydrate matrix (Sigma-Aldrich) in 70% methanol using the SunCollect sprayer (Sunchrom). The matrix was applied in 8 passes (ascending flow rates of 10 μL/min, 20 μL/min, and 30 μL/min for layers 1–3; 40 μL/min for layers 4–8). The MALDI-MSI measurements were performed in negative-ion mode on a 7 T Solarix XR Fourier-transform ion cyclotron resonance mass spectrometer (Bruker Daltonik) equipped with a dual electrospray ionization–MALDI (ESI-MALDI) source and a SmartBeam-II Nd:YAG (355 nm) laser. Mass spectra were acquired over a mass range of m/z 75–1100. The instrument was calibrated externally with L-arginine in the ESI mode and internally using the 9-AA matrix ion signal (m/z 193.0771) as a lock mass. The laser operated at a frequency of 1000 Hz using 100 laser shots per pixel with a spatial resolution of 50 μm.

After MALDI-MSI, the tissue sections were stained with hematoxylin-eosin and picrosirius red, and scanned with an AxioScan.Z1 digital slide scanner (Carl Zeiss) equipped with a 20× magnification objective. The visualization and export of the images to TIFF were done with the software ZEN 2.3 blue edition (Carl Zeiss). MALDI mass spectra were root mean square normalized using SCiLS Lab (Bruker). Metabolites were annotated based on accurate mass matching of 4 ppm or less to the KEGG Pathway Database (https://www.genome.jp/kegg/)^56^ and the Human Metabolome Database (http://www.hmdb.ca/)^57^, allowing [M-H]⁻, [M-H₂O]⁻, [M + K-2H]⁻, [M+Na-2H]⁻, and [M+Cl]⁻ as negative adducts. For glycan annotation, GlycoWorkbench (version 2.1) was used^58^.

### Spatial metabolomics data analysis

For the spatial metabolomics analysis, mass spectrometry imaging (MSI) data from both *Fgf23*^CKO^ and *Fgf23*^fl/fl^ samples were processed using the MALDIquant^37^ package in R. After importing the raw .imzML files, to improve data quality, preprocessing steps including baseline correction, total ion current (TIC) normalization, and peak detection were applied to each spectrum. Peaks were identified using a signal-to-noise ratio (SNR) threshold, and their distributions were examined to ensure spectral consistency between the groups. After preprocessing, the detected peaks were aligned across spectra to enable reliable comparisons.

To identify key metabolic differences between samples, statistical analysis was conducted on the processed data. t-tests with Bonferroni correction were applied to compare peak intensities, highlighting metabolite peaks with adjusted p-values below 0.05. This approach ensured the selection of statistically significant metabolic features, which were further analyzed for biological relevance. These peaks were subsequently annotated using the Human Metabolome Database (HMDB)^59^ for further characterization.

### Spatial transcriptomics

Heart cryosections were air-dried, fixed in methanol at −20 °C, and stained with hematoxylin-eosin (HE) according to standard 10x Genomics protocols. Prior to hybridization, high-resolution images of the HE-stained sections were taken. Spatial transcriptomics were performed according to standard procedures at the Genomics Core Facility of the Medical University Vienna, using the mouse 10× Genomics Fresh-frozen v2 kit on a CytAssist machine.

### Spatial transcriptomics data preprocessing and bioinformatic analysis

For the spatial transcriptomics data analysis, the gene expression matrices and spatial images for *Fgf23*^CKO^ and *Fgf23*^fl/fl^ samples were processed separately using the Seurat package in R^31^. The gene expression data were extracted from HDF5 files, while the spatial images were loaded independently to retain spatial metadata. After ensuring the quality of both sample datasets (Supplementary Fig. S7), they were then combined into Seurat objects through the Load10X_Spatial function. The processed objects were saved as RDS files for further analysis.

After data import, preprocessing steps including normalization, selection of highly variable features, and scaling were applied to both *Fgf23*^CKO^ and *Fgf23*^fl/fl^ samples to ensure data consistency. Metadata annotations were added to distinguish the two groups, and the datasets were merged into a unified Seurat object. The JoinLayers function was used to align data layers, and sample identities were assigned accordingly. Differential gene expression analysis was performed using FindMarkers to identify genes with significant expression differences between the two conditions. A subset of differentially expressed genes was selected based on an adjusted p-value threshold of 0.05 and expression in at least 20% of cells in either group. The resulting gene list was saved for subsequent biological interpretation.

### Functional enrichment analysis

GO (Gene Ontology) enrichment analysis, conducted with Enrichr web tool^60^ against differentially expressed genes, such as comprehensive biological process, cellular component, and molecular function databases, elucidated the functional landscapes of spatially variable genes, identifying potential biological functions of observed spatial expression patterns.

### Statistical analysis of in vivo data

Statistical analysis of the in vivo data was performed using GraphPad Prism 9. The data were analyzed by two-tailed *t*-test for two groups, or by one-way analysis of variance (ANOVA) followed by Student–Newman–Keuls multiple comparison test when comparing more than two groups. Normal distribution of the data was tested by Kolmogorov–Smirnov test. Mass spectrometry data were analyzed using one-way ANOVA followed by Tukey’s test for more than two groups, or by two-tailed *t*-test for two groups. *P*-values of less than 0.05 were considered significant. Data are presented as mean ± SEM or box plots. The indicated *n* values in the graphs refer to the number of animals (biological replicates).

## Supplementary information

Supplementary Material

## Data Availability

All mouse phenotyping data generated or analyzed during this study are included in this published article (and its supplementary information files). The mass spectrometry and spatial transcriptomics data that support the findings of this study are available at 10.5281/zenodo.17256817.
