# Supplementary material for: Deletion of cardiac fibroblast growth factor-23 beneficially impacts myocardial energy metabolism in left ventricular hypertrophy
Source: NPJ Metab Health Dis. 2025 Oct 28;3:42. doi: 10.1038/s44324-025-00087-w (PMC12569085; doi:10.1038/s44324-025-00087-w)
Supplement: Supplementary file 1 — Supplementary Material [file 44324_2025_87_MOESM1_ESM.pdf]

Supplementary Material

Deletion of Cardiac Fibroblast Growth Factor-23 Shifts Myocardial Energy Metabolism Towards a More Beneficial Phenotype in a Left Ventricular Hypertrophy Model

Nejla Latic[1¶], Arezou Lari[2¶], Na Sun[3], Ana Zupcic[1], Mhaned Oubounyt[4], Juliana Falivene[5], Achim Buck[3], Martin Hofer[6], Wenhan Chang[7], Wolfgang M. Kuebler[4], Jan Baumbach[5], Axel K. Walch[4], Alexander Grabner[8#], Reinhold G. Erben[1, 2#*]

[1] Department of Biological Sciences and Pathobiology, University of Veterinary Medicine, Vienna, Austria

[2] Ludwig Boltzmann Institute of Osteology, Vienna, Austria

[3] Research Unit Analytical Pathology, German Research Center for Health and Environment, Neuherberg, Germany

[4] Institute for Computational Systems Biology, University of Hamburg, Hamburg, Germany

[5] Institute of Physiology, Charité University Medicine, Berlin, Germany

[6] Genomics Core Facility, VetCore, University of Veterinary Medicine, Vienna, Austria

[7] San Francisco VA Medical Center, Department of Medicine, University of California, San Francisco, USA

[8] Department of Medicine, Duke University Medical Center, Durham, USA

[¶] These authors share the first authorship

[#] These authors share the last authorship

*** Corresponding author**:

Reinhold G. Erben, M.D., D.V.M.

Ludwig Boltzmann Institute of Osteology, Heinrich-Collin-Strasse 30, 1140 Vienna, Austria

Phone +43-1-910 2186924, E-mail Reinhold.Erben@lbg.ac.at

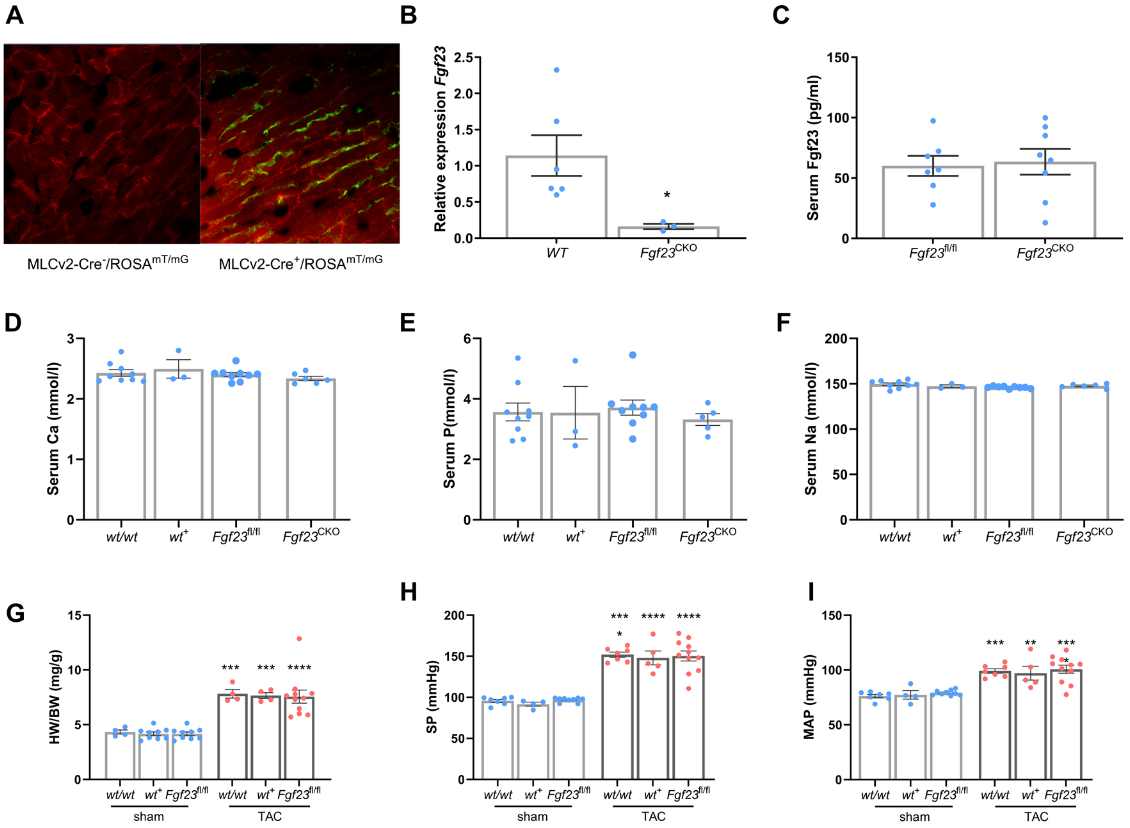

Suppl. Figure S1. Phenotype of mice with a specific deletion of *Fgf23* in cardiomyocytes. (A) Representative images of Cre expression in cardiomyocytes after crossing Mlc2v-Cre mice with ROSA$^{mT/mG}$ reporter mice (scale bar: 20µm). (B) Cardiac relative mRNA expression of *Fgf23* is reduced by 80% in *Fgf23* conditional knock-out (*Fgf23*^{CKO}) mice in comparison to wildtype (wt/wt) mice (n=3-6). (C) Serum intact Fgf23 levels measured by ELISA are comparable between *Fgf23* floxed (*Fgf23*$^{fl/fl}$) and *Fgf23*^{CKO} mice (n = 7-8). (D-F) Serum calcium (Ca), phosphorus (P) and sodium (Na) concentration are comparable in wt/wt, wt Cre^{+} (wt^{+}), *Fgf23*$^{fl/fl}$ and *Fgf23*^{CKO} mice, suggesting that there is no perturbation of systemic mineral metabolism after deletion of *Fgf23* in cardiomyocytes (n=3-9). (G) Heart/body weight ratio, (H) systolic pressure (SP), and (I) mean arterial pressure (MAP) are equally increased in all genotypes after transverse aortic constriction (TAC) (n=4-10). Bars in (A-I) represent mean ± SEM for wt/wt, wt^{+}, *Fgf23*$^{fl/fl}$, *Fgf23*^{CKO} mice after sham and TAC surgery (G-I). *p<0.05, **p<0.01, ***p<0.001, **** p<0.0001 vs sham control by one-way ANOVA followed by Student-Newman-Keuls post-hoc test or by Student's t test for comparison of 2 groups.

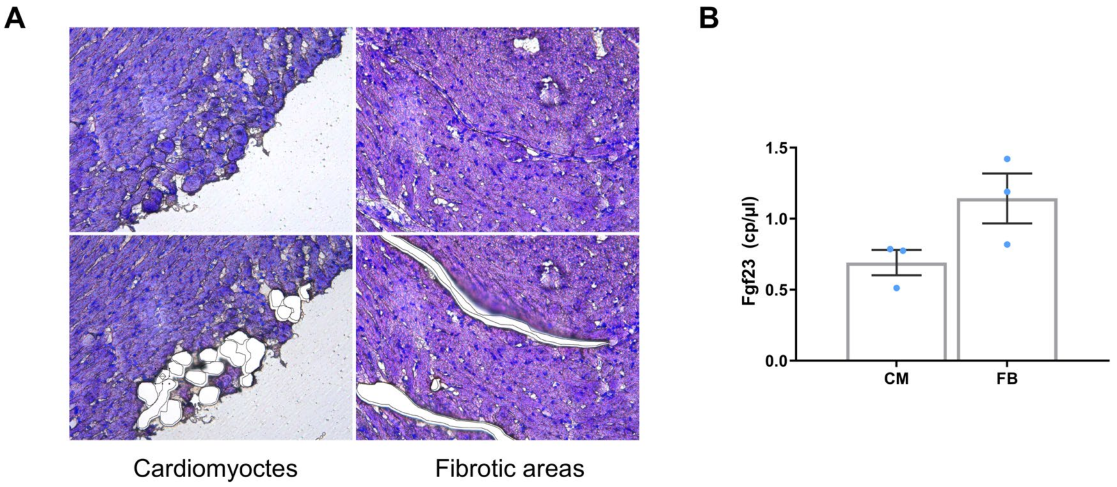

Suppl. Figure S2. Laser capture microdissection reveals comparable Fgf23 expression in cardiomyocytes and fibrotic tissue after TAC. (A) Representative images of cryosections used for laser capture microdissection (LCM), before (upper panels) and after LCM (lower panels) (scale bar: 100 µm). (B) Quantification of *Fgf23* expression by ddPCR in cardiomyocytes and fibrotic areas harvested by LCM revealed comparable numbers of *Fgf23* transcripts in both tissue components (n=3). Bars represent the mean ± SEM.

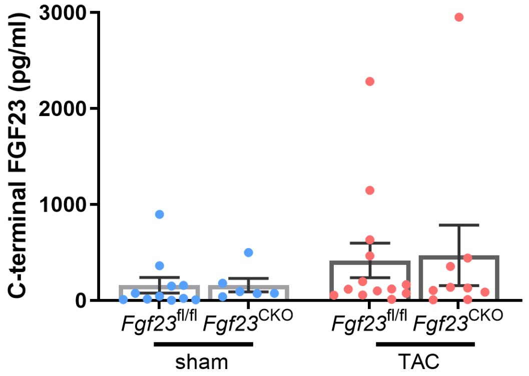

Suppl. Figure S3. Serum C-terminal FGF23 levels in Sham and TAC mice with a specific deletion of *Fgf23* in cardiomyocytes. Serum C-terminal FGF23 levels are comparable between *Fgf23*$^{fl/fl}$ and *Fgf23*^{CKO} mice and between TAC and sham-operated groups (n=6-13).

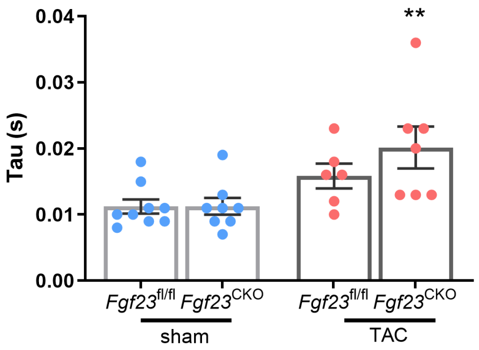

Suppl. Figure S4. Diastolic function assessed by cardiac catheterization in Sham and TAC mice with a specific deletion of *Fgf23* in cardiomyocytes. Cardiac catheterization revealed an increase in Tau in *Fgf23*^{CKO} TAC relative to Sham control mice, suggesting impaired diastolic function in these animals (n=6-9). **p<0.01 vs sham control by one-way ANOVA followed by Student-Newman-Keuls post-hoc test.

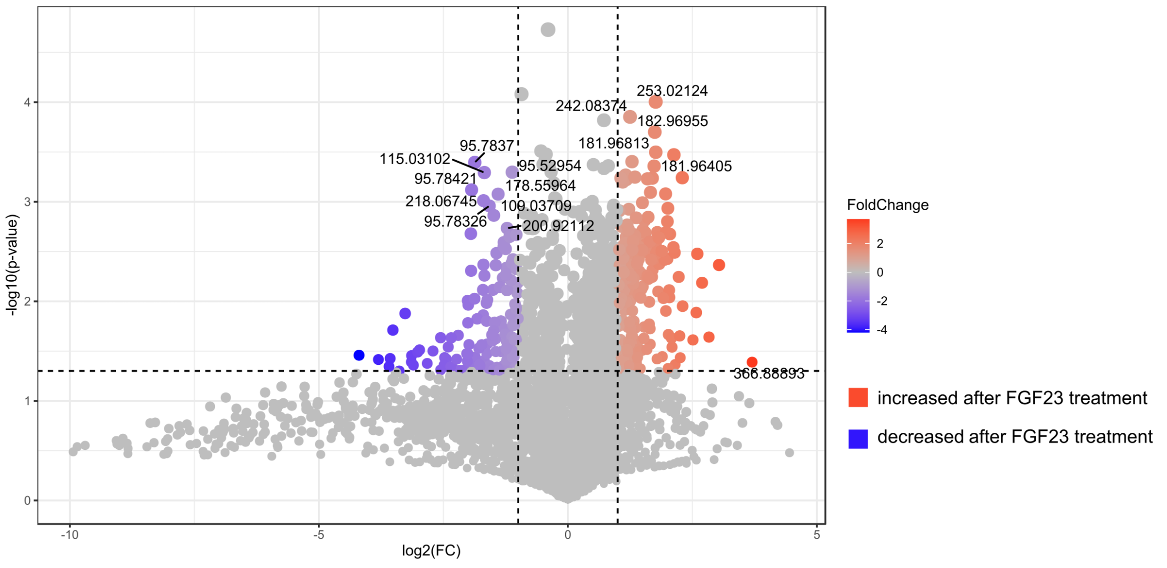

Suppl. Figure S5. Volcano plot displaying the analysis of metabolites by MALDI imaging in FGF23-treated cultured cardiomyocytes. Volcano plot showing that treatment of cultured rat neonatal cardiomyocytes with rFGF23 for 48 h induces distinct changes in metabolic signature, relative to vehicle-treated cells. The volcano plot combines fold change (FC) and t-tests, with the x-axis representing fold change (FC) and the y-axis representing log10 p-value. Discriminative metabolites are selected using the volcano plot criteria: fold change > 2 or < 0.5 and a p-value < 0.05. Metabolites that are increased after FGF23 treatment are shown in red, those decreased after FGF23 treatment are shown in blue, and grey spots indicate unchanged metabolites.

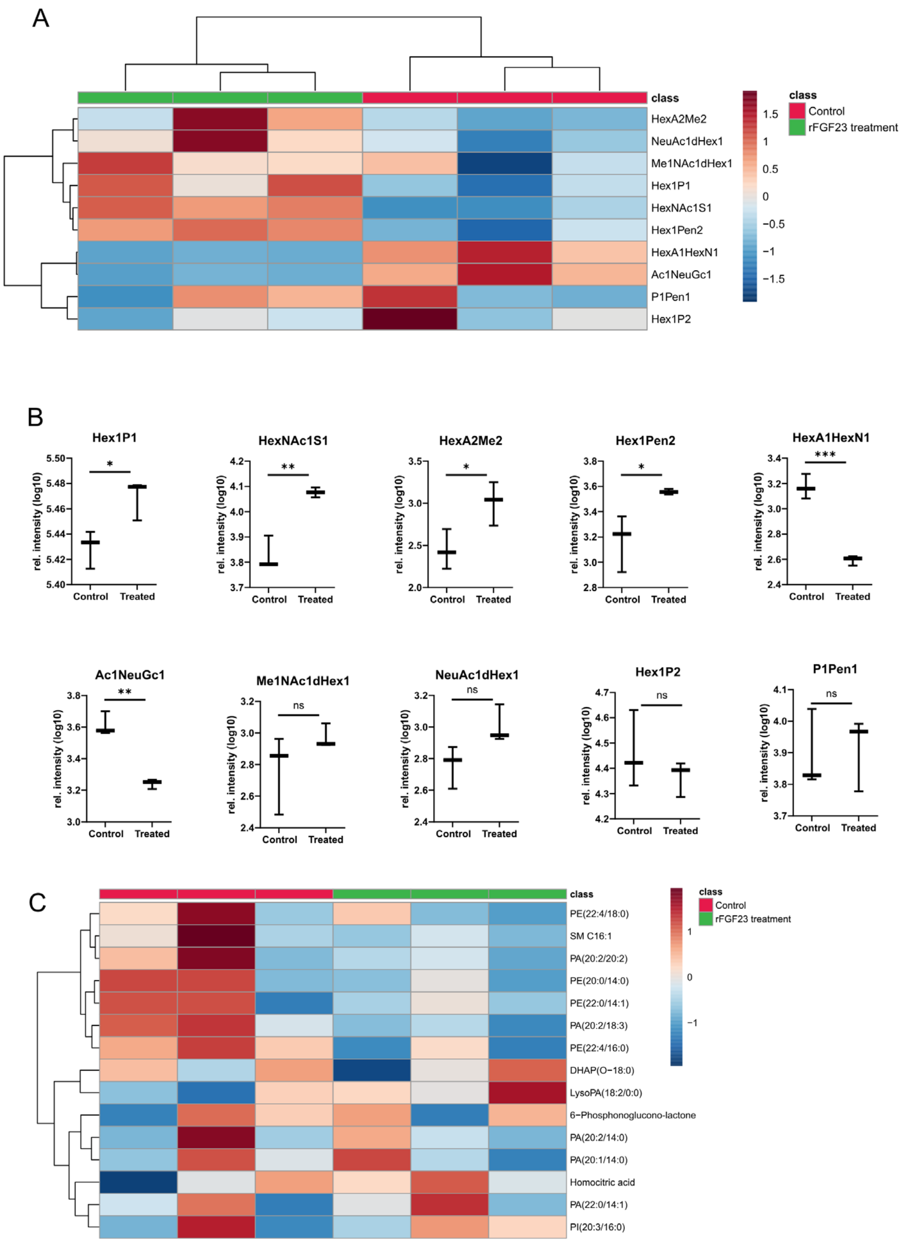

Suppl. Figure S6. MALDI imaging-based clustering analysis of glycans and lipids in FGF23-treated cultured cardiomyocytes. (A) Heatmap of glycans shows distinct changes in metabolic glycan signature in cultured neonatal rat cardiomyocytes induced by rFGF23 treatment compared to vehicle-treated cells. (B) Hex1P1, HexNac1S1, HexA2Me2, and Hex1Pen2 exhibit increased abundance, whereas the abundance of HexA1HexN1 and Ac1NeuGc1 is reduced in rFGF23-treated, relative to the vehicle-treated cardiomyocytes. The glycans Me1NAc1dHex1, NeuAc1dHex1, Hex1P2, and P1Pen1, remain unchanged under FGF23 treatment. (C) Heatmap of lipids shows that treatment of cultured cardiomyocytes with rFGF23 induces changes in lipid pattern compared to vehicle-treated cells. *p<0.05; **p<0.01; ***p<0.001; ns, not significant by two-tailed t test.

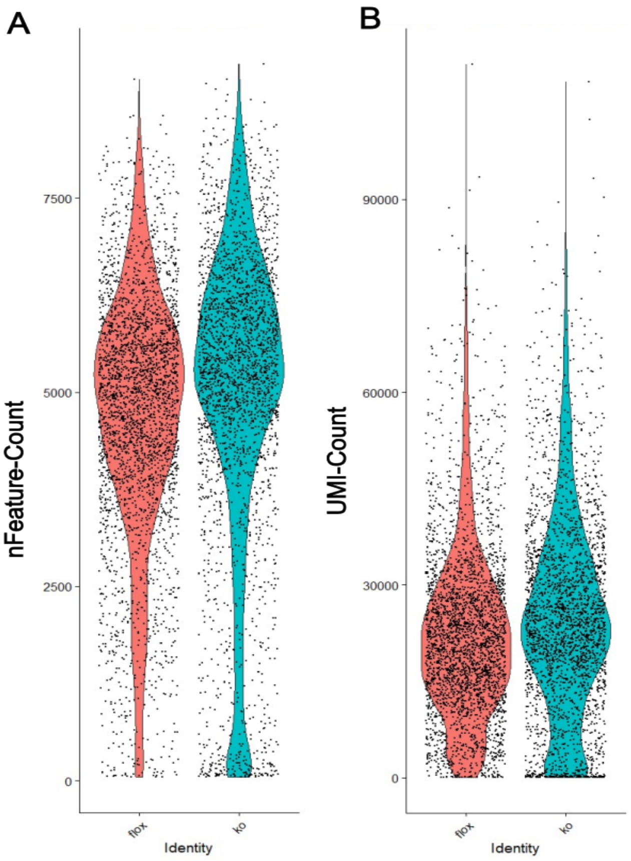

Suppl. Figure S7. Violin plots showing quality control metrics for floxed and KO samples from Visium spatial transcriptomics. (A) The plot shows that the number of genes detected in each spot (nFeature_Spatial) was similar between the two groups. (B) The total number of transcripts captured per spot (UMI counts) also shows a similar distribution across both samples, with no major outliers.

Suppl. Table 1– Mouse primer sequences for quantitative real-time PCR analysis

Gene	Forward (5´-3´)	Reverse (5´-3´)
Bnp	GCC AGT CTC CAG AGC AAT TCA	GCC ATT TCC TCC GAC TTT TCT
Anp	GGC CAT ATT GGA GCA AAT CCT	GCA GGT TCT TGA AAT CCA TCA GA
